# Exploring the Perceived Value of Standing in Individuals with Lower Limb Impairments

**DOI:** 10.3390/jcm14145161

**Published:** 2025-07-21

**Authors:** Yukiyo Shimizu, Hideki Kadone, Yosuke Eguchi, Kai Sasaki, Kenji Suzuki, Yasushi Hada

**Affiliations:** 1 Department of Rehabilitation Medicine, Institute of Medicine, University of Tsukuba, Tsukuba 305-8575, Japan; y-hada@md.tsukuba.ac.jp; 2 Department of Cybernics Medicine, Institute of Medicine, University of Tsukuba, Tsukuba 305-8575, Japan; kadone@md.tsukuba.ac.jp; 3 Qolo Inc., Tsukuba 305-8577, Japan; eguchi@qolo.jp (Y.E.); kai.addaption@gmail.com (K.S.); 4 Institute of Systems, Information and Engineering, University of Tsukuba, Tsukuba 305-8575, Japan; kenji@ieee.org

**Keywords:** assistive technology, lower limb impairment, psychosocial benefit, rehabilitation, sedentary behavior, spinal cord injury, standing

## Abstract

**Background:** Standing has medical and psychosocial benefits for people with lower limb impairments; however, systemic, logistical, and economic barriers often limit opportunities to stand in daily life. This study explored how users perceive standing and standing-assistive technologies. **Methods**: This study used a mixed-methods approach: in-person interviews (*n* = 18) and a nationwide web-based survey (*n* = 125; 74.4% male, mean age 52.2 ± 13.9 years, diagnoses: spinal cord injury 37.6%, cerebrovascular disease 27.2%, and cerebral palsy 16.8%). **Results**: Participants described the psychosocial values of standing, such as feeling more confident and being able to interact with others at eye level. The web survey revealed that most participants believed that standing was beneficial for health (76.8%) and task efficiency (76.0%), although only 49.6% showed an interest in standing wheelchairs. The multivariate analysis revealed that ongoing standing training was the strongest predictor of positive perceptions of health benefits, task efficiency, and interest in standing wheelchairs. Younger participants showed a greater interest in standing wheelchairs. The reported barriers include a lack of awareness, high costs, and difficulty in accessing training. **Conclusions:** These findings suggest the need for a user-centered design and improved support systems to integrate standing into the daily lives of people with mobility impairments.

## 1. Introduction

Wheelchairs are indispensable for individuals with trunk and lower limb impairments when performing daily activities. However, these individuals are often required to remain seated for extended periods. Prolonged sitting increases the risk of physical complications, including contractures of the hip and knee joints, pressure ulcers, and reduced cardiovascular and digestive systems [1]. Additionally, eye-level disparities and limited environmental accessibility create social and functional challenges [2,3]. These physical and social limitations may contribute to psychosocial challenges, although research specifically examining users’ perspectives on standing remains limited.

Standing position has several well-documented medical benefits. Clinical guidelines recommend standing exercises for individuals with spinal cord injuries (SCIs) [4]. The reported benefits include maintaining bone mineral density, preserving joint range of motion, reducing spasticity, enhancing the quality of life, regulating bowel function, stimulating the cardiovascular and respiratory systems, and preventing pressure ulcers [4].

Despite these advantages, standing exercises are often difficult to implement in individuals with lower limb impairments. Tilt-table training is effective but is typically restricted to specialized facilities. Standing while using orthoses is another option. However, these devices are expensive and difficult to wear. In Japan, these challenges are exacerbated by the national healthcare system, which limits insurance-covered rehabilitation to 180 days after physical impairment onset. Rehabilitation is provided in 20 min units, up to a maximum of 180 min per day, with authorized durations ranging from 90 to 180 days depending on the diagnosis and clinical evidence [5]. Although this structure facilitates acute and subacute care, it tends to limit access to long-term or maintenance interventions such as standing training. In addition, the lack of continuity between medical and long-term care systems in Japan restricts consistent rehabilitation in the chronic phase [6].

In recent years, sedentary behavior has been linked to health risks in able-bodied individuals. Even among those capable of standing, a sedentary lifestyle has been associated with increased risks of cardiovascular events, sleep disorders, and mortality [7,8,9]. Furthermore, since the onset of the coronavirus disease (COVID-19) pandemic, remote work and online meetings have become increasingly common, leading to a more sedentary lifestyle in the general population. In this context, the long-term health effects of prolonged sitting have become a matter of growing concern [10,11].

Individuals with SCI have been reported to have an increased risk of atherosclerosis [12]. This may be attributed not only to the direct physiological consequences of the injuries themselves but also to extended periods of sitting [13].

Motivated by both the clinical benefits and implementation challenges of standing, we developed a non-powered, gas-spring-assisted standing mobility device designed to support upright movements in everyday life. We specifically chose this design to enable users with lower limb impairments to initiate standing independently according to their intentions and timing. This device, named Qolo, enables users to perform sit-to-stand transitions using gas-spring assistance and to move in a standing posture via a powered wheelchair base [14]. Although the concept was based on medical engineering principles, we questioned whether it truly aligned with the expectations and values of potential users. Specifically, we wondered whether the importance of standing was genuinely shared by users or whether it merely reflected the assumptions of the developers.

To investigate this, we conducted in-person interviews and a nationwide web-based survey of individuals with lower limb impairments to better understand their perspectives on standing and standing-related technologies.

This study sought to reconsider the significance of standing by exploring how individuals who are generally assumed to be unable to stand because of lower limb impairment perceive the possibility of standing again.

## 2. Materials and Methods

We conducted a mixed-methods study comprising two independent components: in-person interviews and a nationwide web-based survey. Each component included a distinct group of participants.

First, in-person interviews were conducted with 18 individuals with trunk and/or lower limb impairments. Participants were included if they were aged 18–74 years, had trunk and/or lower limb impairments requiring wheelchair use, and had prior experience with the Qolo standing device. Individuals with cognitive impairments that would prevent informed consent or those who could not communicate in Japanese were excluded. Individual and small-group formats were used in this study. The primary aim was to obtain qualitative insights into how these individuals perceived the role of standing and upright mobility in their daily lives.

Ethical approval for in-person interviews was obtained from the Institutional Review Board of the Faculty of Engineering, Information, and Systems at the University of Tsukuba (approval no. 2019R300-2, approved on 3 September 2020). Written informed consent was obtained from all participants before data collection.

For the web-based survey, participant recruitment and data collection were conducted using a marketing platform operated by DO HOUSE, Inc. (Tokyo, Japan; currently Excrie Inc.). In total, 69,998 individuals responded to the initial screening, of whom 1593 reported having been issued a physical disability certificate by the Japanese government. From this group, 125 individuals were included in the final analysis based on the following criteria: aged 18–74 years, current or previous wheelchair use owing to trunk and/or lower limb impairments, and ability to complete the survey in Japanese.

The sample size for the interviews (*n* = 18) was determined through the purposive sampling of eligible participants with relevant experiences. We employed a pragmatic sampling strategy using an online research platform to achieve a sample size of 125.

The web survey consisted of 15 questions covering demographics (five items), wheelchair use patterns (three items), standing training experience (four items), and perceptions of standing (three items: perceived health benefits, perceived task efficiency, and standing wheelchair interest/ownership; see Supplementary Material S4 for the exact wording). The response format included multiple-choice and open-ended text fields. The semi-structured interviews included eight open-ended questions that focused on daily experiences, standing situations, and technology perceptions. Additionally, the survey respondents were asked whether they were interested in a standing wheelchair without being provided any information about the Qolo we were developing. The web-based survey was conducted anonymously and no personally identifiable information was collected.

Statistical analyses were performed using the JMP Student Edition 18 (SAS Institute Inc., Cary, NC, USA). Descriptive statistics were used to summarize the participant characteristics. Chi-square (χ^2^) tests were applied to compare nominal variables, and Fisher’s exact test was employed when expected cell counts were small. Student’s *t*-test was used to compare continuous variables between groups. One-way analysis of variance (ANOVA) was conducted to compare continuous variables, such as age and wheelchair use duration, across the diagnosis groups.

Logistic regression analyses were conducted to examine the factors associated with the following three binary outcomes: interest in standing wheelchairs, perceived health benefits of standing, and perceived task efficiency while standing. The independent variables entered into all the models were age, sex, primary diagnosis, employment status, wheelchair use duration, wheelchair use frequency, and standing training experience. All the variables were simultaneously included in the multivariate model without variable selection. Statistical significance was defined as *p* < 0.05.

Reference categories were determined using statistical software defaults, except for the primary diagnosis, where cerebral palsy was retained as the reference because of its congenital nature and distinct clinical trajectory compared to acquired conditions. Sensitivity analyses were conducted using alternative reference categories to confirm the robustness of the findings.

## 3. Results

We conducted in-person interviews with 18 individuals with physical or neurological impairments, including SCI, cerebral palsy, and neuromuscular disorders. The participants ranged in age from 18 to their 50s and included seven employed individuals, four students, and four homemakers. All participants had prior experience in trialing a Qolo standing mobility device.

The interview data revealed a consistent recognition of the value of standing in everyday life. Participants frequently mentioned the importance of interacting with others at eye level, particularly during presentations, conversations while walking, and shared activities such as lectures and meetings. Standing has also been viewed as beneficial for functional independence in both domestic and public settings.

Shopping was frequently mentioned as a situation in which standing provided significant benefits. Several participants reported difficulty in accessing items on high shelves and discomfort associated with asking for assistance. They described instances in which they requested help to retrieve a product, only to receive an unintended item. Owing to their reluctance to repeatedly request assistance, the participants often ended up purchasing something different or abandoning the purchase. Standing mobility was defined as the ability to regain the ability to inspect and select items independently, allowing for more convenient and confident shopping experiences.

Regarding the web survey, a total of 125 individuals with impairments of the trunk or lower limbs who used a wheelchair were included in the final analysis (Table 1). Among the participants, 93 were male (74.4%) and 32 were female (25.6%). The mean age of the participants was 52.2 ± 13.9 years (range: 24–72 years).

Employment status varied; 62 participants (49.6%) were employed, 8 (6.4%) engaged in housework, and 55 (44.0%) were unemployed. With respect to the regions of impairment (multiple responses allowed), 103 participants reported lower limb impairments, 57 reported upper limb impairments, and 47 reported trunk impairments. As wheelchair use was an inclusion criterion, those with upper limb impairments also had concurrent trunk and/or lower limb impairments.

The primary diagnoses were SCI (*n* = 47; 37.6%), cerebrovascular disease (*n* = 34; 27.2%), and cerebral palsy (*n* = 21; 16.8%). The remaining 23 individuals (18.4%) had other neurological disorders. The mean duration of wheelchair use was 14.8 ± 13.0 years. Regarding usage frequency, 84 participants (67.2%) reported daily wheelchair use, whereas 41 (32.8%) reported using a wheelchair several times per week.

To explore the differences across diagnostic groups, we first examined the associations between the primary diagnosis and demographic/clinical characteristics (Table 2). Significant differences were found in age and wheelchair use duration, with individuals with cerebrovascular disease being older on average (58.1 ± 12.3 years, *p* = 0.0126) and those with cerebral palsy reporting the longest duration of wheelchair use (24.0 ± 16.0 years, *p* < 0.0001).

Regarding the standing training status, 55 participants (44.0%) were currently undergoing training, 36 (28.8%) had discontinued their previous training, and 34 (27.2%) had no experience with standing training. The diagnostic group distributions differed across these training status categories. Among those with no experience (*n* = 34), 14 had SCI, 4 had cerebrovascular disease, 8 had cerebral palsy, and 8 had other diagnoses. In the discontinuation group (*n* = 36), 18 patients had SCI, 9 had cerebrovascular disease, 6 had cerebral palsy, and 3 had other conditions. Among those currently continuing training (*n* = 55), 15 had SCI, 21 had cerebrovascular disease, 7 had cerebral palsy, and 12 had other diagnoses.

A statistically significant association was observed between the training status and diagnosis (Fisher’s exact test, *p* = 0.0363). Notably, 61.8% of participants with cerebrovascular disease were currently undergoing training, compared to only 31.9% with SCI and 33.3% with cerebral palsy.

Among participants with experience in standing training (*n* = 91), hospitals or clinics were the most common (54.5%), followed by home-based training (33.3%) and other medical or rehabilitation facilities (12.1%). Among those currently undergoing training (*n* = 55), the proportion of participants who were training at home was higher (51.0%), whereas fewer participants were trained at hospitals, clinics (31.0%), or other facilities (18.0%) (Table 3).

Among the 55 participants currently undergoing standing training, the majority (*n* = 30, 54.5%) practiced almost daily, 19 (34.5%) trained several times per week, 5 (9.1%) trained once per week, and 1 (1.8%) trained once or twice per month. Standing training methods varied among the participants, including long-leg braces (36.3%), standing frames or tilt tables (27.5%), and manual training without devices (33.0%), confirming that the participants referred to conventional rehabilitation methods rather than specific standing wheelchair products (see Supplementary Material S4 for details).

The most frequently cited reason for discontinuation (*n* = 36) was improvement in standing ability (13 participants, 36.1%), followed by difficulty in accessing medical institutions (10, 27.8%), difficulty wearing orthoses (7, 19.4%), lack of anticipated recovery (3, 8.3%), and other reasons (3, 8.3%) (Table 4). Of the 13 patients who reported improvement, 7 had cerebrovascular disease, 4 had SCI, and 2 had neurodegenerative conditions; no participants with cerebral palsy were included in this group. Among those who cited a lack of anticipated recovery, two had SCI and one had cerebrovascular disease.

Table 5 presents the three standing-related outcomes stratified by the primary diagnosis. Overall, 96 participants (76.8%) believed that standing had health benefits and 95 (76.0%) felt that standing enhanced task efficiency. However, only 62 individuals (49.6%) expressed an interest in using a standing wheelchair. Significant differences were found across the diagnostic groups for task efficiency (*p* = 0.0314) and interest in standing wheelchairs (*p* = 0.0386), whereas perceived health benefits showed a marginal difference (*p* = 0.0638).

Participants with cerebrovascular disease showed the highest agreement regarding health benefits (88.2%) and task efficiency (88.2%), whereas interest in standing wheelchairs was lowest in this group (32.4%). By contrast, participants with other diagnoses showed the highest interest in standing wheelchairs (69.6%).

Among the 125 respondents, 21 (16.8%) currently owned a standing wheelchair, including 3 with cerebral palsy (14.3%), 8 with SCI (17.0%), 3 with cerebrovascular disease (8.8%), and 7 with other diagnoses (30.4%). A significant difference in ownership was found across diagnostic groups (Fisher’s exact test, *p* < 0.001), with the other diagnoses group showing notably higher ownership rates. For the analysis of interest in standing wheelchairs, we combined those who owned a standing wheelchair (*n* = 21) with those who did not own one but expressed interest (*n* = 41), resulting in 62 participants (49.6%) categorized as interested, while 63 (50.4%) were not interested. The levels of interest differed significantly between the diagnostic groups (chi-square test, *p* = 0.0386). Only 32.4% of the participants with cerebrovascular disease showed interest, compared to 48.9% with SCI, 57.1% with cerebral palsy, and 69.6% with other diagnoses.

We conducted exploratory comparisons of mean age and wheelchair use duration between the outcome groups (Supplementary Table S1). While no consistent patterns were observed, participants who expressed interest in standing wheelchairs were significantly younger (49.1 ± 15.4 years) than those who did not (55.3 ± 11.5 years; *p* = 0.0123).

The open-ended responses from those who answered affirmatively revealed several common themes. The most frequently cited benefits were reaching elevated objects (*n* = 20), performing various tasks (*n* = 12), bathing or showering (*n* = 4), improving the visual field (*n* = 4), smoother transfers (*n* = 4), psychological benefits (*n* = 4), performing tasks in elevated places (*n* = 3), shopping (*n* = 3), and work-related activities (*n* = 2) (Table 6).

Participants who expressed no interest in standing wheelchairs (*n* = 63) were asked to select all the applicable reasons for not choosing standing-function products. The most common reasons were lack of awareness of the product and perceived lack of need (both *n* = 26, 41.3%). Notably, 14 participants (22.2%) reported that they could stand independently with bilateral support and therefore did not require assistance. Practical barriers included high cost and vehicle incompatibility (both *n* = 12, 19.0%), as well as concerns about device size (*n* = 6, 9.5%). Critically, one participant reported that no standing wheelchairs existed that accommodated their specific physical needs, highlighting the limitations of the current product designs (Table 7).

Multivariate logistic regression analyses were performed to examine the following factors associated with the binary outcomes: perceived health benefits of standing, perceived task efficiency during standing, and interest in standing wheelchairs (Table 8). The independent variables were age, sex, primary diagnosis, employment status, duration and frequency of wheelchair use, and standing training experience. All variables were entered simultaneously without stepwise selection. Statistical significance was defined as *p* < 0.05.

The models revealed the following significant associations: For perceived health benefits of standing, younger age (OR = 0.941 per year, 95% CI: 0.896–0.983, *p* = 0.0086) and ongoing standing training (OR = 6.815, 95% CI: 1.867–24.880, *p* = 0.0037) were significant predictors. For perceived task efficiency during standing, only ongoing standing training was significantly associated (OR = 8.338, 95% CI: 2.253–30.857, *p* = 0.0015), with marginal effects observed for wheelchair use duration (OR = 1.042, 95% CI: 0.999–1.090, *p* = 0.0608) and daily wheelchair use frequency (OR = 2.611, 95% CI: 0.869–7.842, *p* = 0.0873). Interest in standing wheelchairs was independently associated with ongoing standing training (OR = 5.362, 95% CI: 1.797–15.992, *p* = 0.0026) and cerebrovascular disease but not cerebral palsy (OR = 6.693, 95% CI: 1.507–29.717, *p* = 0.0124).

Multicollinearity among independent variables was assessed using the variance inflation factor (VIF). All VIF values were below 5 (range: 1.6–2.5), indicating that multicollinearity was not a concern in our models. The highest correlations were observed between age and diagnosis, which reflects the expected association between age and disease type (e.g., cerebrovascular disease occurring predominantly in older adults).

To examine the robustness of our findings, we conducted sensitivity analyses using different reference categories as the primary diagnostic variables. Regardless of the chosen reference category, the pattern of associations remained consistent, with participants with cerebrovascular disease showing the lowest interest in standing wheelchairs across all comparisons (Supplementary Table S2).

## 4. Discussion

This study reconsidered the significance of standing in daily life by exploring the perspectives of individuals with lower limb impairments. Although we asked about task efficiency, many participants emphasized psychological benefits such as feeling more confident, emotionally uplifted, or socially included. This finding illustrates that standing is not merely a functional action but also a meaningful psychosocial experience, challenging assumptions about why individuals with mobility impairments value standing. Similar findings have been described in previous studies, in which standing was associated with improved self-esteem and social interactions in individuals with SCI [4].

Clinically, standing has been shown to help preserve joint range of motion, prevent contractures and pressure ulcers, reduce spasticity, and stimulate the cardiovascular, respiratory, and gastrointestinal systems [1,15]. Despite these benefits, access to standing remains limited owing to logistical and financial constraints. For instance, in Japan, time-limited insurance coverage and short rehabilitation durations contribute to the low continuity of long-term standing interventions [6].

Our multivariate logistic regression analysis showed that ongoing standing training was the strongest predictor of all three outcomes. People currently performing standing training had significantly higher odds of perceiving health benefits (OR = 6.815), task efficiency (OR = 8.338), and interest in standing wheelchairs (OR = 5.362). This finding was consistent regardless of diagnosis, age, or other factors. This suggests that active experience with standing training may be more important than personal characteristics in shaping positive attitudes toward standing. The high percentage perceiving health benefits (76.8%) indicates that users value standing not only for immediate practical advantages but also potentially for functional recovery, consistent with evidence that motor training can promote neuroplasticity in neurorehabilitation [16,17,18,19].

Notably, the health consequences of prolonged sitting are not limited to individuals with disabilities. A growing body of evidence demonstrates that sedentary behavior in the general population is associated with an increased risk of cardiovascular diseases, type 2 diabetes, and all-cause mortality [20,21,22]. These findings suggest that standing, both as a posture and behavioral strategy, has substantial physiological significance, even for able-bodied individuals. By illustrating the multifaceted value of standing for individuals with mobility impairment who require specialized technologies, this study also calls attention to the underappreciated health and psychosocial implications of prolonged sitting times. This reinforces the need to re-evaluate sedentary patterns in daily life across all populations and not just among those with physical impairments.

Differences between the diagnostic groups were also observed. In our bivariate analysis, individuals with cerebrovascular disease showed the least interest in standing wheelchairs (32.4%). The multivariate analysis initially appeared contradictory, showing higher odds than cerebral palsy (OR = 6.693). However, this finding resulted from our choice of a reference category. When we conducted sensitivity analyses using different reference categories, we found that participants with cerebrovascular disease consistently showed the lowest interest in standing wheelchairs compared to all other diagnostic groups (Supplementary Table S2). The initial bivariate finding was partially confounded by age and standing training status; patients with cerebrovascular disease were older and more likely to be undergoing standing training (61.8%). After accounting for these factors, the lower interest in standing wheelchairs became more apparent. This may reflect their relatively preserved walking function or intermittent wheelchair use, suggesting that the perceived need for assistive devices is strongly shaped by individual functional capacity and daily context, likely reflecting their acquired disability status, relatively preserved walking function, and different recovery expectations compared to those with congenital or traumatic conditions. In contrast, younger individuals and those with early onset disabilities such as SCI or cerebral palsy showed greater interest, possibly due to greater long-term motivation or adaptation to technological aid. These findings indicate that age, diagnosis timing, and disability onset patterns strongly influence enthusiasm for standing mobility devices.

Our findings reveal an important gap between recognizing the benefits of standing and the interest in standing wheelchairs. Although over 75% acknowledged the health and efficiency benefits, only half expressed interest in standing wheelchairs. This disconnect highlights that perceived benefits alone are insufficient for technology adoption and that practical barriers must also be addressed.

Qualitative data also highlighted the situational benefits of standing, shopping, presentations, and reaching high places, where an upright posture enables more autonomy and participation. However, users simultaneously cited real-world barriers such as difficulty accessing rehabilitation facilities and the discomfort or complexity of orthotic devices. These practical challenges must be addressed along with technological developments to ensure real-life usability.

Despite these challenges, recent studies have demonstrated that standing wheelchairs can be safely used by individuals with SCI [23], with reported benefits, including improved bowel regularity and reduced spasticity [24]. These effects are further supported by evidence of enhanced pressure redistribution in SCI [25] and improved bone density and joint range in children with neuromuscular disorders [26]. Moreover, these functional gains are consistent with user-reported priorities, including restored independence and improved quality of life [23], as further evidenced by successful long-term community integration studies of affordable manual standing wheelchairs [27,28].

Taken together, our findings align with the WHO’s 5Ps framework for assistive technology [29]. The diverse needs across diagnostic groups (People), varying interest in standing wheelchairs (Product), cost barriers (Policy), limited access to training facilities (Provision), and importance of ongoing professional support (Personnel) demonstrate that improving access to standing technologies requires comprehensive strategies addressing all five components. This comprehensive approach is crucial for improving both the functional independence and quality of life of wheelchair users.

This study has several limitations. First, qualitative interviews were conducted with a relatively small group of participants, all of whom had prior trial experiences with the standing wheelchair Qolo. The participants were recruited through hospital demonstrations, representing a potentially more motivated subset of wheelchair users. Their enthusiasm for standing technology may not reflect the views of the broader wheelchair-using population, particularly those who are skeptical of new technologies or satisfied with their current mobility solutions. This selection bias limits the generalizability of our qualitative findings, although the web-based survey with a broader sample helps to partially address this limitation. Second, the web-based survey relied on self-reported data, which may have introduced a recall bias. Third, the participants were recruited from an online panel rather than random sampling, which may have skewed the data toward those who were more digitally engaged and health-conscious. The 16.8% standing wheelchair ownership rate in our sample may reflect this selection bias toward individuals with greater motivation for standing. Additionally, we acknowledge that our sample size, particularly for multivariate analyses with 10 predictors, may have been underpowered according to conventional guidelines. However, the consistency of findings across different analytical approaches and the exploratory nature of this study provide valuable preliminary insights that can inform future confirmatory research with larger sample sizes. Finally, the diagnostic classifications were broad, and uncontrolled variables such as the duration of disability and rehabilitation history likely influenced the participants’ perspectives.

Future research should incorporate objective functional evaluations, refine diagnostic subgroups, and include diverse populations to improve generalizability. Despite these limitations, this study provides valuable insights into the perceived value of standing and highlights the need for comprehensive strategies for clinical, technological, and policy development.

## 5. Conclusions

Standing is valued by many individuals with lower limb impairments not only for functional gains but also for its psychosocial significance. However, enthusiasm for standing-assistive technologies varies depending on the patient’s age, diagnosis, and lifestyle. While the clinical benefits of standing are well recognized, the practical and perceptual barriers remain unresolved. Effective adoption requires a user-centered approach that considers individual differences in physical function, motivation, and daily activity requirements.

## Figures and Tables

**Table 1:** Participant characteristics and standing-training-related variables (*N* = 125).

Variable	Response	*n*	%
Sex	Male	93	74.4
	Female	32	25.6
Age (Years)	Mean ± SD	52.2 ± 13.9	—
Employment	Employed	62	49.6
	Housework	8	6.4
	Unemployed	55	44.0
Region of Impairment	Lower limbs	103	—
	Upper limbs	57	—
	Trunk	47	—
Primary Diagnosis	Cerebral palsy	21	16.8
	Spinal Cord Injury	47	37.6
	Cerebrovascular diseases	34	27.2
	Other	23	18.4
Wheelchair Use	Duration (years) ± SD	14.8 ± 13.0	—
Frequency	Daily	84	67.2
	Several times/week	41	32.8
Standing Training Status	Ongoing	55	44.0
	Discontinued	36	28.8
	No Experience	34	27.2

Categorical variables are presented as frequencies (*n*) or percentages (%). Continuous variables are reported as mean ± standard deviation (SD). Variables were grouped according to demographic characteristics, wheelchair use, impaired regions, primary diagnosis, and standing-training-related attributes.

**Table 2:** Participant characteristics and standing training status by primary diagnosis (*N* = 125).

Variable	Cerebral Palsy (*n* = 21)	Spinal Cord Injury (*n* = 47)	Cerebrovascular Disease (*n* = 34)	Other (*n* = 23)	*p*-Value (Test)
Sex (Male)	13 (61.9%)	34 (72.3%)	30 (88.2%)	16 (69.6%)	0.1155 (Fisher)
Employment (Employed)	12 (57.1%)	27 (57.4%)	12 (35.3%)	11 (47.8%)	0.2668 (Fisher)
Mean Age (years)	49.0 ± 14.6	51.9 ± 11.6	58.1 ± 12.3	47.0 ± 17.0	0.0126 (ANOVA) *
Duration of Wheelchair Use (years)	24.0 ± 16.0	18.8 ± 13.8	8.6 ± 5.9	7.3 ± 5.9	<0.0001 (ANOVA) *
Wheelchair Use (Daily)	14 (66.7%)	38 (80.9%)	20 (58.8%)	12 (52.2%)	0.0542 (Chi-square)
Standing Training					0.0363 (Fisher) *
Ongoing	7 (33.3%)	15 (31.9%)	21 (61.8%)	12 (52.2%)	
Discontinued	6 (28.6%)	18 (38.3%)	9 (26.5%)	3 (13.0%)	
No Experience	8 (38.1%)	14 (29.8%)	4 (11.8%)	8 (34.8%)	

Values are shown as numbers (percentage) or mean ± standard deviation, unless otherwise noted. *p*-values are reported for comparisons across the primary diagnosis groups; * *p* < 0.05.

**Table 3:** Primary training locations among participants with standing training experience and those currently training status by diagnosis.

Group	Home (%)	Hospital/Clinic (%)	Other Facilities (%)
Any Training Experienced (*n* = 91)	33.3	54.5	12.1
Currently Training (*n* = 55)	51.0	31.0	18.0

**Table 4:** Reasons for discontinuing standing training (*n* = 36).

Reason	*n* (%)
Improved standing ability	13 (36.1%)
Difficulty accessing medical institutions	10 (27.8%)
Difficulty in wearing orthoses	7 (19.4%)
Lack of anticipated recovery	3 (8.3%)
Other reasons	3 (8.3%)

**Table 5:** Standing-related outcomes by primary diagnosis.

Outcome	Response	Overall (*n* = 125)	Cerebral Palsy (*n* = 21)	Spinal Cord Injury (*n* = 47)	Cerebrovascular Disease (*n* = 34)	Other (*n* = 23)	*p*-Value (Test)
Perceived Health Benefits	Yes	96 (76.8%)	12 (57.1%)	35 (74.5%)	30 (88.2%)	19 (82.6%)	0.0638 (Fisher)
	No	29 (23.2%)	9 (42.9%)	12 (25.5%)	4 (11.8%)	4 (17.4%)	
Perceived Task Efficiency	Yes	95 (76.0%)	11 (52.4%)	36 (76.6%)	30 (88.2%)	18 (78.3%)	0.0314 (Fisher) *
	No	30 (24.0%)	10 (47.6%)	11 (23.4%)	4 (11.8%)	5 (21.7%)	
Interest in Standing Wheelchairs	Yes	62 (49.6%)	12 (57.1%)	23 (48.9%)	11 (32.4%)	16 (69.6%)	0.0386 (Chi-square) *
	No	63 (50.4%)	9 (42.9%)	24 (51.1%)	23 (67.6%)	7 (30.4%)	

Distribution of “Yes” and “No” responses for three standing-related outcomes by primary diagnosis, *n* (%). Fisher’s exact test was used to assess group differences, except for interest in standing wheelchairs (chi-square test). Participants with cerebrovascular disease showed the highest agreement regarding health benefits and task efficiency, whereas interest in standing wheelchairs was the lowest in this group. * *p* < 0.05.

**Table 6:** Reported efficiency improvements from standing (*n* = 95).

Reported Benefit	Number of Responses
Reaching high objects	20
Performing various activities	12
Taking a bath or shower	4
Improved visual field	4
Transfers	4
Improved mood	4
Tasks performed in elevated places	3
Shopping	3
Work	2

**Table 7:** Reasons for lack of interest in standing wheelchairs (*n* = 63).

Reason	*n*	%
Lack of product awareness	26	41.3
Perceived lack of need	26	41.3
Can stand independently with support, no assistance needed	14	22.2
High cost	12	19.0
Incompatibility with vehicles (difficult to load/transport)	12	19.0
Device appears too large and inconvenient	6	9.5
Fear or perceived danger of standing	3	4.8
Time-consuming standing process	3	4.8
Other	1	1.6
Total responses	103	

**Table 8:** Multivariable logistic regression analysis for standing-related outcomes.

Independent Variables	Perceived Health Benefits	Perceived Task Efficiency	Interest in Standing Wheelchairs
	OR (95% CI)	OR (95% CI)	OR (95% CI)
Age (per year)	0.941 (0.896–0.983) *	0.965 (0.920–1.008)	1.008 (0.974–1.044)
Gender (ref: Female)			
Male	1.722 (0.549–5.399)	0.863 (0.256–2.909)	1.065 (0.365–3.108)
Primary Diagnosis (ref: Cerebral Palsy)			
Spinal Cord Injury	0.606 (0.179–2.048)	0.428 (0.122–1.505)	1.949 (0.580–6.548)
Cerebrovascular Disease	0.514 (0.099–2.660)	0.427 (0.079–2.295)	6.693 (1.507–29.717) *
Other	0.315 (0.055–1.788)	0.501 (0.089–2.814)	1.037 (0.226–4.761)
Employment Status (ref: Unemployed)			
Employed	2.279 (0.744–6.977)	1.317 (0.438–3.963)	1.543 (0.622–3.826)
Housework	0.947 (0.076–11.762)	0.779 (0.054–11.325)	0.611 (0.087–4.292)
Wheelchair Use Duration (per year)	1.020 (0.979–1.064)	1.042 (0.999–1.090)	1.022 (0.985–1.062)
Wheelchair Use Frequency (ref: Several times/week)			
Daily	1.550 (0.523–4.598)	2.611 (0.869–7.842)	1.512 (0.615–3.715)
Standing Training Status (ref: No Experience)			
Ongoing	6.815 (1.867–24.880) *	8.338 (2.253–30.857) *	5.362 (1.797–15.992) *
Discontinued	3.690 (0.944–14.433)	3.272 (0.835–12.828)	2.632 (0.921–7.520)

All models were adjusted for age, sex, primary diagnosis, employment status, duration and frequency of wheelchair use, and standing training status. Reference categories were determined using statistical software defaults, except for the primary diagnosis, where cerebral palsy was retained as the reference because of its congenital nature and distinct clinical trajectory compared to acquired conditions. Statistically significant *p*-values (*p* < 0.05) are marked with asterisks (*).

## Data Availability

The original contributions presented in this study are included in the article. Further inquiries can be directed to the corresponding author.

## Supplementary Materials

The following supporting information can be downloaded at: https://www.mdpi.com/article/10.3390/jcm14145161/s1.
