# Supplementary material for: Exploring the Perceived Value of Standing in Individuals with Lower Limb Impairments
Source: J Clin Med. 2025 Jul 21;14(14):5161. doi: 10.3390/jcm14145161 (PMC12296183; doi:10.3390/jcm14145161)
Supplement: Supplementary file 1 [file jcm-14-05161-s001.zip › jcm-3750232-supplementary.pdf]

Journal of Clinical Medicine

MDPI

Supplementary Table S1. Age and Duration of Wheelchair Use in Response to Standing Outcomes

Outcome	Variable	Yes	No	p-value
Perceived Health Benefits	Mean Age (years)	53.4 ± 13.3	48.0 ± 15.3	0.0653
	Wheelchair Use Duration (years)	13.0 ± 12.4	16.6 ± 13.5	0.114
Perceived Task Efficiency	Mean Age (years)	52.8 ± 14.0	50.4 ± 13.5	0.4106
	Wheelchair Use Duration (years)	13.1 ± 12.4	16.0 ± 13.7	0.2053
Interest in Standing Wheelchairs	Mean Age (years)	49.1 ± 15.4	55.3 ± 11.5	*0.0123*
	Wheelchair Use Duration (years)	13.0 ± 13.8	16.6 ± 12.1	0.114

Comparison of mean age and wheelchair use duration between "Yes" and "No" response groups for each standing-related outcome. Values are expressed as mean ± standard deviation. P-values were obtained using unpaired t-tests. These analyses were exploratory and not part of the primary regression model. Statistically significant p-values (P < 0.05) are marked with asterisks (*).

Supplementary Table S2. Odds Ratios for Interest in Standing Wheelchairs with Different Reference Categories

Primary Diagnosis	Reference: Cerebral Palsy	Reference: SCI	Reference: Cerebrovascular Disease
Cerebral Palsy	1.00 (ref)	0.513 (0.153-1.724)	0.291 (0.090-0.940)*
SCI	1.949 (0.580–6.548)	1.00 (ref)	0.149 (0.034-0.663)*
Cerebrovascular Disease	6.693 (1.507–29.717)*	3.434 (1.063-11.09)*	1.00 (ref)
Other	1.037 (0.226–4.761)	0.532 (0.149-1.901)	0.155 (0.040-0.595)*

*p < 0.05

Supplementary Table S3. Key Survey Questions and Analysis Categories

Domain	Original Question (Translated from Japanese)	Response Options	Analysis Categories
Interest in Standing Wheelchairs	"Do you own a wheelchair with a standing function? Please select the most appropriate answer:"	1. Yes, I own one (n=21); 2. No, I don't own one, but I am interested (n=41); 3. No, I don't own one and I am not interested (n=63)	Interested: Options 1+2 (n=62, 49.6%); Not interested: Option 3 (n=63, 50.4%)

Perceived Health Benefits	"When you encounter statements like 'Continuing standing training contributes to health maintenance and improvement,' which best describes your perception?"	1. Strongly agree; 2. Agree; 3. Disagree; 4. Strongly disagree	Yes: Options 1+2 (n=96, 76.8%); No: Options 3+4 (n=29, 23.2%)
Perceived Task Efficiency	"Are there situations where you feel standing improves task efficiency?"	1. Yes; 2. No	Yes (n=95, 76.0%); No (n=30, 24.0%)

Note: We examined whether ownership of standing wheelchairs differed by diagnosis using Fisher's exact test due to small cell sizes and found no significant differences (p=0.140), supporting our decision to analyze overall interest regardless of ownership status.

Supplementary Material S4: Complete Web Survey Questionnaire and Results

Section 1: Demographic and Disability Characteristics

Q1. Sex

- Male: 93 (74.4%)
- Female: 32 (25.6%)

Q2. Age

- Mean ± SD: 52.2 ± 13.9 years
- Range: 24-72 years

Q3. Employment Status

- Employed: 62 (49.6%)
- Unemployed: 55 (44.0%)
- Housework: 8 (6.4%)

Q4. Physical Disability Certificate Type (multiple responses allowed)

- Lower limbs: 103
- Upper limbs: 57
- Trunk: 47

Q5. Primary Diagnosis

- Spinal cord injury: 47 (37.6%)

- Cerebrovascular disease: 34 (27.2%)
- Cerebral palsy: 21 (16.8%)
- Other: 23 (18.4%)

Section 2: Wheelchair Use Patterns

Q6. Duration of wheelchair use

- Mean ± SD: 14.8 ± 13.0 years

Q7. Frequency of wheelchair use

- Daily: 84 (67.2%)
- Several times per week: 41 (32.8%)

Section 3: Standing Training Experience

Q8. Have you ever performed standing training?

- Yes (currently continuing): 55 (44.0%)
- Yes (discontinued): 36 (28.8%)
- No: 34 (27.2%)

Q9. For those currently training, what is your training frequency?

- Nearly daily: 30 (54.5%)
- Several times per week: 19 (34.5%)
- Once per week: 5 (9.1%)
- 1-2 times per month: 1 (1.8%)
- Several times per year: 0 (0.0%)

Q10. For those who discontinued treatment, what was the main reason?

- Recovery of standing ability: 13 (36.1%)
- Difficulty accessing training facilities: 10 (27.8%)
- Difficulty with preparation (e.g., putting on orthoses): 7 (19.4%)
- Other: 6 (16.7%)

Q11. Main training locations (for those with training experience).

- Hospital/clinic: 39 (42.9%)
- Home: 35 (38.5%)
- Other facilities: 17 (18.7%)

Q12. For those with training experience, what type of equipment or training methods did you use/did you use?**

- Long-leg braces/knee-ankle-foot orthoses: 33 (36.3%)
- Standing frames/tilt tables: 25 patients (27.5%)
- Manual training without devices 30 (33.0%)
- Other: 3 (3.3%)

Section 4: Perceptions of Standing

Q13. When you encounter statements like "Continuing standing training contributes to health maintenance and improvement," which best describes your perception?

- Heard about it and agree: 67 (53.6%).
- Have never heard about it but agree: 29 (23.2%)
- Have never heard about it and disagree: 18 (14.4%)
- Heard about it and disagree: 11 (8.8%)

Q14. Are there situations in which you feel standing improves task efficiency?

- Yes: 95 (76.0%)
- No: 30 (24.0%)

Section 5: Standing Wheelchair Interest

Q15. Do you own a wheelchair with a standing function?

- Yes: 21 (16.8%)
- No, and not interested: 63 (50.4%)
- No, but interested: 41 (32.8%)

Q16. For those not interested, reasons for not choosing standing wheelchairs (multiple responses allowed, n=63)

- Lack of product awareness: 26 (41.3%)
- Perceived lack of need: 26 (41.3%)
- Can stand independently with support; no assistance needed: 14 (22.2%)
- High cost: 12 (19.0%)
- Incompatibility with vehicles (difficult to load/transport): 12 (19.0%)
- Device appears too large and inconvenient: 6 (9.5%)
- Fear or perceived danger of standing: 3 (4.8%)
- Time-consuming standing process: 3 (4.8%)
- Other*: 1 (1.6%)
- Total responses: 103

*The participant who selected "Other" specified that no standing wheelchairs exist that accommodate their specific physical needs.
